# Companion Diagnostics for Targeted Cancer Drugs – Clinical and Regulatory Aspects

**DOI:** 10.3389/fonc.2014.00105

**Published:** 2014-05-16

**Authors:** Dana Olsen, Jan Trøst Jørgensen

**Affiliations:** ^1^Regulatory Affairs, Dako Denmark A/S, an Agilent Technologies Company, Glostrup, Denmark; ^2^Dx-Rx Institute, Fredensborg, Denmark

**Keywords:** companion diagnostics, *in vitro* diagnostics, drug–diagnostic co-development, regulatory requirements, personalized medicine, precision medicine, oncology

## Abstract

Companion diagnostics (CDx) holds the promise of improving the predictability of the oncology drug development process and become an important tool for the oncologist in relation to the choice of treatment for the individual patient. A number of drug–diagnostic co-development programs have already been completed successfully, and in the clinic, the use of several targeted cancer drugs is now guided by a CDx. This central role of the CDx assays has attracted the attention of the regulators, and especially the US Food and Drug Administration has been at the forefront in relation to developing regulatory strategies for CDx and the drug–diagnostic co-development project. For an increasing number of cancer patients the treatment selection will depend on the result generated by a CDx assay, and consequently this type of assay has become critical for the care and safety of the patients. In order to secure that the CDx assays have a high degree of analytical and clinical validity, they must undergo an extensive non-clinical and clinical testing before release for routine patient management. This review will give a brief introduction to some of the scientific and medical challenges related to the CDx development with specific emphasis on the regulatory requirements in different regions of the world.

## Introduction

The understanding of the molecular mechanisms of cancer has increased considerably within the last 10–20 years, which has resulted in the development of a number of new targeted drugs. A large proportion of these drugs has been developed using the drug–diagnostic co-development model where the diagnostic test and the drug are developed in parallel (1, 2). The use of this model requires a thorough understanding of the underlying molecular pathology and the drug mechanisms of action, in order to link a certain molecular characteristic to the treatment outcome. The first attempt to use the drug–diagnostic co-development model was made when trastuzumab (Herceptin^®^, Roche/Genentech) and a immunohistochemistry (IHC) assay were developed for HER2 positive advanced breast cancer (3, 4). Since the approval of trastuzumab and the IHC assay for HER2 overexpression (HercepTest™, Dako) in 1998 by the US Food and Drug Administration (FDA), a number of new targeted cancer drugs guided by a diagnostic assay, a companion diagnostic (CDx) test, has been approved and introduced in the clinic to the benefit of the patients (5). The importance of incorporating a CDx in a drug research project has recently been emphasized by the fact that approximately two-thirds of the breakthrough therapy designations granted by the FDA include a diagnostic assay (6).

The main purpose of developing a CDx assay in most oncology drug research programs is to have a test that can predict whether a patient is likely to benefit from the drug in question. Hence, for many targeted cancer drugs the CDx assays will take up a central role as a kind of “decisive” stratification factor, both during development and subsequently after approval when the drug is used in the clinic. The assay will then become a kind of “gatekeeper” in relation to the treatment decision (2). However, if a CDx assay measures a specific biomarker or combination of biomarkers and it turns out that it is not sufficiently correlated with the clinical state, which could be overexpression of a specific protein or genetic mutations, it will not provide meaningful results. Such an erroneous test result could lead to either a false positive or false negative result, which potentially may cause risk and harm to the patient. For example, a false positive result could lead to treatment with a drug where the biological condition for a positive outcome is missing, and consequently the patient is put at risk due to potential toxic side effects from an ineffective treatment. Similarly, a false negative test result could withhold or delay a potentially beneficial treatment and thereby also bringing the patient at risk (7). In oncology, an early and correct diagnosis and intervention are two elements of key importance in the treatment of cancer patients. In case of a wrong treatment decision, the disease may become disseminated with no or very low chances of cure (2).

The central role of CDx assays in relation to both drug development and the clinical use after approval has caught the attention of the regulatory authorities. Especially the FDA has been at the forefront in relation to developing regulatory strategies for drug–diagnostic co-development and personalized medicine. As described above, it is important to avoid false positive and false negative test results and the analytical and clinical validity of any CDx assay must be sufficiently documented before it can be approved for routine use in the clinic (1, 7). In this article some of the scientific and medical challenges related to the CDx development are discussed with specific emphasis on the regulatory requirements.

## Companion Diagnostics – Terminology and Definitions

With regards to the terminology and definitions of a diagnostic assay that is developed in parallel to a targeted drug and used to guide the treatment decision, there seems to be lack of consensus. Different names are used in the literature, such as pharmacodiagnostics, theranostics, pharmacogenomic biomarkers, and companion diagnostics. Within the last few years, the name companion diagnostics has been used more and more frequently and this is also the term that has been adapted by the FDA and now also the European Union (EU), however, theranostics is still used quite frequently especially in the academic literature (2). In 2011, the FDA issued a draft guidance on *In vitro Companion Diagnostics Devices* where a CDx was defined (8). According to this definition a CDx assay is an *in vitro* diagnostic device that provides information that is essential for the safe and effective use of a corresponding therapeutic product. Further, the FDA specifies three areas where a CDx assay is essential: (1) to identify patients who are most likely to benefit from a particular therapeutic product; (2) to identify patients likely to be at increased risk of serious adverse reactions as a result of treatment with a particular therapeutic product; and (3) to monitor response to treatment for the purpose of adjusting treatment (e.g., schedule, dose, discontinuation) to achieve improved safety or effectiveness. So according to the FDA, a CDx assay can be used both to predict outcome (efficacy and safety) and to monitor the response.

The definition that has been proposed by the EU is somewhat narrower and is more or less limited to item 1 in the FDA definition. According to the proposed regulation on *in vitro* diagnostic medical devices from 2012, a CDx is a device specifically intended to select patients with a previously diagnosed condition or predisposition as eligible for a targeted therapy (9). With no doubt the predictive or selective characteristics of a CDx assay has so far attracted the most attention. The use of a CDx assay facilitates the design of clinical trials with a smaller number of subjects, which has a positive effect on the resources and time spent on clinical development (2). A definition that focuses on the predictive or selective characteristics of the CDx assay and makes a link to “personalized medicine” is: “A pre-treatment test performed in order to determine whether or not a patient is likely to respond to a given therapy. This type of test is classified as a predictive or selective test and is a prerequisite for implementation of personalized and stratified medicine” (10).

## Drug–Diagnostic Co-Development

**Figure 1:**
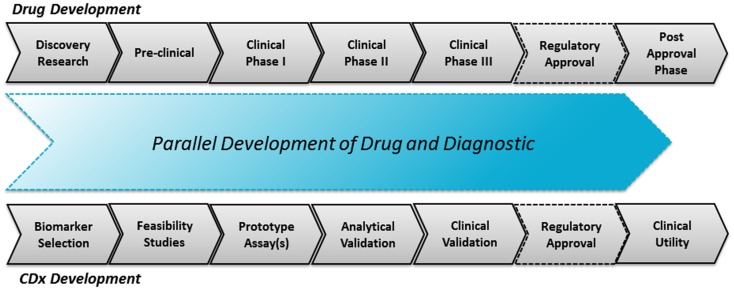
**The drug–diagnostic co-development model**. The upper parts illustrate the drug development process and the lower parts the parallel CDx development process with an aligned regulatory co-approval at the end of phase III.

In the drug–diagnostic co-development model there is interdependency of drug and diagnostics. The CDx assay is developed in parallel to the drug, as illustrated in Figure 1. The success of such a co-development project depends very much on the strength of the biomarker hypothesis, which is often deduced during the early research and preclinical phases of the drug development. As previously mentioned, it requires a thorough molecular understanding of both the pathology and drug mechanisms of action to come up with a solid hypothesis. It might not only be one hypothesis which is tested through prototype assays but several hypotheses. These prototype assays are subsequently used during the clinical phases I and II in order to give an idea of the predictive potential.

If one or more of these hypotheses appears promising the assay will then undergo analytical validation. However, before the analytical validation of the CDx assay can be finalized, the cut-off value must be established, which is usually done based on outcome data from phase I/II clinical trials. During the analytical validation, it must be demonstrated that the assay accurately and reliably measures the biomarker that has been selected earlier on in the development process. In relation to this validation, a number of both internal and external studies must be performed. For the external analytical validation a multi-site study is performed to document reproducibility using the final version of the CDx assay across several laboratories. Before using the CDx assay for patient selection and treatment stratification in a clinical phase III trial, it is strongly recommended that the assay is analytically validated (1, 7). Due to challenges with respect to the alignment and timing of the development of the drug and the CDx assay, it is sometimes tempting to start the clinical trial with a prototype assay and then replace it with the validated version later on during the trial. However, such a strategy is not recommendable as it makes it difficult to interpret the clinical trial results due to the fact that the patients have been selected using two different versions of the assay (7). If different versions of an assay have been used during clinical validation a subsequent bridging study will be needed, which is both resource demanding and time consuming. A “golden rule” with regards to the final clinical validation of a CDx is to use only one version of the assay, which is the analytically validated version, and only one testing laboratory in order to reduce possible site to site variation.

In the drug–diagnostic co-development model, phase III is not only used to demonstrate safety and efficacy of the drug, but also to clinically validate the CDx assay. Here, it must be demonstrated that the CDx assay has an ability to predict the treatment outcome in the individual patients (7). A CDx assay will only be useful if it provides information that can discriminate between patients who are likely responders and non-responders, and in this respect the clinical diagnostic accuracy of the assay is important, thus data on the clinical sensitivity, specificity, positive predictive value (PPV), and negative predictive value (NPV) for the CDx assay are important diagnostic metrics to consider. Several trial designs for clinical drug–diagnostic co-development have been proposed, however, not all of them make it possible to calculate the described diagnostic metrics. Table 1 provides a brief overview of the main clinical trial designs that have been proposed for the parallel development of drug and diagnostic, however, in this article only the enrichment design will be discussed, as it is the design that so far has been used most frequently in relation to drug–diagnostic co-development. Furthermore, a relatively large number of review articles and draft guidance document have been published within the last few years describing these trial designs in more details (1, 2, 11–14).

**Table 1:** **Overview of the main clinical trial designs that have been proposed for the parallel development of drugs and diagnostics**. The last column in the table lists the diagnostic metrics that can be calculated based on the given clinical trial design. CDx+, test positive patients; CDx−, test negative patients; PPV, positive predictive value; NPV, negative predictive value.

Clinical trial design	Description	Diagnostic metrics
All-comers*	All patients meeting the study eligibility criteria are enrolled in the trial independent of the CDx test results	Sensitivity, specificity, PPV, and NPV
Enrichment	Only patients who are CDx+ and meet the study eligibility criteria are enrolled in the trial	PPV
Stratified	Both CDx+ and CDx−patients meeting the study eligibility criteria are enrolled in the trial and subsequently randomized	Sensitivity, specificity, PPV, and NPV

*Low prevalence of CDx+ patients requires a large sample size.

**Figure 2:**
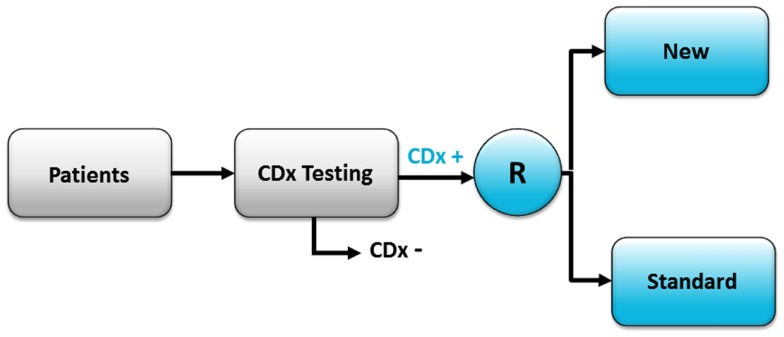
**The enrichment clinical trial design**. With this design only patients who have a positive CDx assay result are enrolled in the trial and randomized (R) to either the new targeted treatment (New) or standard treatment (Standard). CDx+, indicates test positive patients; CDx−, indicates test negative patients.

The enrichment trial design is often used if there is clear evidence of a strong relationship between a positive CDx status and the treatment outcome with the targeted drug (e.g., from previous phase I/II studies) (1, 2). With this design, all the patients are tested by means of a CDx assay, but only the CDx positive patients are enrolled in the study and subsequently randomized to either the new targeted drug or to the standard treatment, as shown in Figure 2. The advantage of this design is that it generally requires a smaller number of patients to be randomized compared with the all-comers design, due to the fact that only patients who have a CDx positive status are enrolled in the trial, thus making the study population more homogeneous. However, this design allows only the PPV to be calculated and not sensitivity, specificity, and NPV, which is a limitation of this a trial design (1, 2). The enrichment design was also the one used when trastuzumab went through final phase III testing in women with advanced breast cancer in the 1990s (3). Further, looking at the drug–diagnostic combinations that have obtained FDA approval, the enrichment design is the most frequently used to demonstrate safety and efficacy of the drug and to clinically validate the corresponding CDx assay. Recent examples of targeted cancer drugs that have used this trial designs are vemurafenib (Zelboraf™, Roche/Genentech), crizotinib (Xalkori^®^, Pfizer), pertuzumab (Perjeta^®^, Roche/Genentech), ado-trastuzumab emtansine (Kadcyla^®^, Roche/Genentech), dabrafenib (Tafinlar^®^, GSK), and trametinib (Mekinist™, GSK). A list of the CDx assays and their corresponding therapeutic product that have been approved by the FDA can be found at the webpage of Center for Devices and Radiological Health (CDRH) (5).

**Figure 3:**
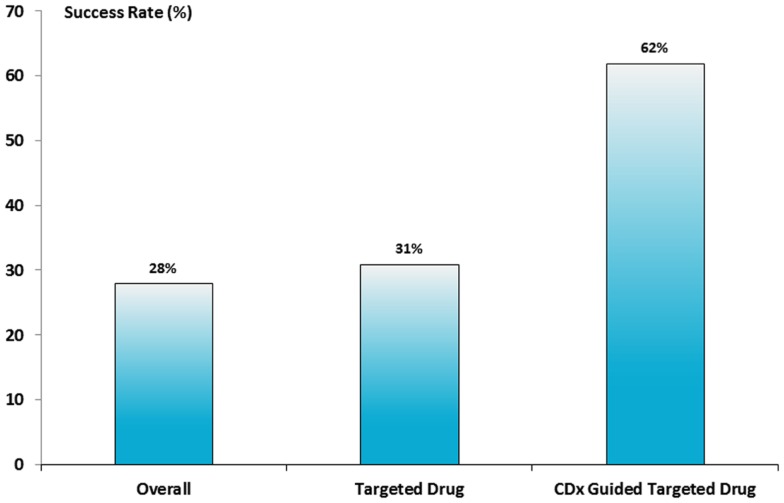
**Success rate for NSCLC drugs in phase III clinical trials**. Based on the analysis of 676 clinical trials a success rate of 28% was found for all types of drugs, however, if the drug was either a receptor target drug or guided by a CDx assay the success rate increased to 31 and 62%, respectively (15).

How effective is the use of a CDx in the drug development process? This question was partly answered in an analysis made to estimate the risk of clinical trial failure during non-small cell lung cancer (NSCLC) drug development in the period between 1998 and 2012 (15). The data material was retrieved from different available public sources and 676 clinical trials with 199 unique drug compounds meeting the inclusion criteria of the analysis. The data showed that the success of clinical phase III was the biggest obstacle for drug approval with an overall success rate of only 28%. A small improvement in the success rate was found for the receptor targeted therapies tested in phase III. However, the absolutely highest success rate was observed when the drug was biomarker-guided showing a success rate of 62%, as seen in Figure 3. So, the conclusion from this analysis indicates that the use of a CDx assay during phase III drug development improves the success rate considerably. The data from this analysis also seem to confirm the effectiveness of the enrichment design described earlier in this paragraph.

## Companion Diagnostics and Regulatory Requirements

Recent developments in the field of personalized medicine and drug–diagnostic co-development have been most challenging, not only for the regulatory professionals but also for regulatory authorities. While drug companies and CDx manufacturers found new grounds in collaboration to jointly bring their products to patients, some regulatory authorities have been too slow to adapt to the changing regulatory landscape caused by the CDx development. A few highlights of the regulatory process for CDx in the major markets are presented and discussed below.

### FDA setting the standard for regulatory pathway of companion diagnostics

In April 2005, the FDA published the Drug–Diagnostic Co-Development Concept Paper. This document labeled by the FDA “Draft Preliminary Concept Paper – Not for Implementation” has become a landmark for the formalization of the drug–diagnostic co-development strategy (1). Even though the pharma and diagnostic companies have seldom found the co-development model for parallel development of drug and diagnostic feasible, it provided grounds for alternative development strategies and for obtaining FDA feedback prior to initiating non-clinical or clinical testing, or prior to intended submission of a marketing application. Since 2005, the FDA has taken the lead and set the standard for the CDx regulatory pathway. This standard also provided inspiration to other authorities and regulatory professionals worldwide. The FDA further strengthened its leading position in defining the regulatory landscape for CDx by creating a personalized medicine group within the Office of *In vitro* Diagnostics and Radiological Health (OIR), formerly, Office of *In vitro* Diagnostics (OIVD) in 2009. This group has contributed to a considerable number of guidance documents related to CDx. Furthermore, FDA is providing transparency of the approval process by including web availability of Safety and Effectiveness Summary documents for the approved CDx.

While waiting for an update of the 2005 concept paper, a draft of the *In vitro* Companion Diagnostic Devices guidance was published in July 2011 (8). This guidance document is not a replacement of the 2005 concept paper, but rather an operational guide for *In vitro* Diagnostics (IVD) of the pharma and biotech industries indicating possible regulatory pathways as well as labeling and regulatory requirements for CDx devices and therapeutic products (5).

Attention should be paid to an important section of the guidance covering the investigational use of CDx. Before the “companion diagnostics era,” many of the investigational IVD devices were either exempted from Investigational Device Exemption (IDE) regulations or classified as non-significant risk devices subject to abbreviated IDE requirements. In the case of clinical trials, where companion diagnostics are used to make a medical decision – such as treatment assignment, an IVD is considered a serious risk device requiring IDE approval by the FDA. Typically, a pharma or a biotech company is the sponsor of a drug–diagnostic clinical trial conducted under the Investigational New Drug (IND) regulations. However, it is important that an IDE for the diagnostic is either included in the IND or submitted and approved separately. According to the guidance document, FDA accepts that the IDE information is included in the IND. However, as the IDE format is not compatible with an IND, in some cases, the FDA has expressed that a separate IDE is preferred (16). Hopefully, this will be further clarified in the final version of the guidance, which is expected by October 2014 (17). The content of an IDE is well-defined in the regulations and further specified on the FDA website and in several guidance documents (18). In the case of a combined drug–diagnostic clinical trial, the IDE must, in addition to information on the CDx assay, also include information provided by the drug sponsor, such as the clinical trial protocol, investigational sites, Investigational Review Board (IRB) information, and informed consent material for patients. Thus, in relation to collaboration between a drug company and a diagnostic company, it is important that roles, responsibilities, and timelines are clearly defined between the parties.

In the 2011 guidance document, the FDA declares that “the FDA review of the test/therapeutic product pair will be carried out collaboratively among relevant FDA offices.” Truly, FDA offices responsible for each of the products are not only collaborating in the review process but are also announcing approvals of both the drug and CDx concurrently.

Another important guidance, not only for CDx, is the Medical Devices: the Pre-Submission Program and Meetings with FDA Staff published in draft in July 2012. Final version of the guidance was published in February 2014 (19). In this document, the Pre-IDE program was renamed to a Pre-Submission (Pre-Sub) program (19). Since 1995, the Pre-IDE, now Pre-Sub, program, has allowed industry to obtain FDA feedback prior to any kind of device submission and thus providing opportunities for the industry to discuss a drug–diagnostic co-development strategy at any development or testing stage. Even though there is no user fee for a Pre-Sub, the process has become more formalized since authorization of the Medical Device User Fee and Modernization Act (MDUFMA) in 2012 (20). In the new guidance, the FDA provides recommendations to the contents of the Pre-Sub and also clarifies the administrative procedures of the program. The Pre-Sub is a formal, written request for feedback from the FDA regarding analytical or clinical study protocols or a proposed regulatory pathway. A Pre-Sub may also be an appropriate way to acquaint the FDA with a novel technology or design. A Pre-Sub interaction with the FDA is a particularly useful way to discuss testing strategies which are not the ideal co-development scenarios, and where an analytically validated assay is not available at an early stage of the clinical drug development. The benefits of Pre-Subs may include time and cost reduction of research or clinical studies, better understanding of FDA expectations and trends, especially in the area where no guidance documents are available, and, most importantly, may result in a better and more complete marketing application and greater chance of a successful approval. In order to improve the understanding of IVD related issues, it is recommended that the drug sponsor participates in the Pre-Sub process initiated by the diagnostic company. If relevant, CDRH will request Center for Drug Evaluation and Research (CDER) attendance in the process. According to the FDA statistics, the inter-center consultations have increased from 39 in 2010 to 106 in 2012 (16), which is a likely consequence of the increased number of drug–diagnostic co-development projects mainly within oncology.

The controls required by the FDA prior to marketing of a device in the US depend on the classification of the device. Medical devices, including IVDs, are risk-classified as class I, II, or III. The majority of companion diagnostic IVDs are high risk class III devices. This review will not go into details of the regulatory requirements for each product class, but only give a very brief summary. For class I devices, general controls, like establishment registration and device listing, apply; for class II devices, general controls and a premarket clearance [510(k)] is needed; and class III devices require the most stringent approval for medical devices by the FDA, a Premarket Approval Application (PMA) (21).

Briefly, a PMA application may be either traditional or modular. There is no difference in the contents of a traditional or a modular PMA but there is a difference in the way the PMA is submitted for FDA review. In a traditional PMA, all information required by the regulations is submitted at the same time, while for a modular PMA the information is submitted in modules. Thus, analytical performance (non-clinical studies) and manufacturing information may be submitted and reviewed by the FDA while a clinical trial is ongoing. When the clinical trial is completed, the data will then be submitted to the FDA. At this point of time, the other modules have been through FDA review. This approach may allow for a shorter approval process and a better alignment with the drug approval.

A modification in an intended use for a PMA-approved CDx, such as adding a new indication or a new targeted drug, is a complex process which, depending on the type of modification, may require massive analytical and/or clinical data. The FDA is very responsive to Pre-Subs for device modifications and provides feedback to proposed regulatory pathways and studies supporting regulatory submission for the change in the intended use of the specific CDx.

### Europe tightens up the IVD legislation

The IVD Directive 98/79/EC regulates *in vitro* diagnostic medical devices in the EU, EU candidate countries, and associated countries (22). The current EU regulatory framework for IVD devices demonstrates how unnoticed CDx IVD devices were at the end of the nineties when the IVD Directive was proposed and subsequently entered into force in 2003. There is no specific mention of CDx in the definition of an IVD, and the classification system of the directive does not consider CDx at all. Also, the IVD Directive list-based classification system has shown its limitations, as only a limited number of IVD devices are considered medium or high risk devices (so-called Annex II devices). All remaining IVDs, including CDx assays, are classified as low risk devices. Adding a new device to the Annex II list has proved to be a cumbersome process. It has taken 4 years to add a variant Creutzfeldt–Jakob disease assay to List A of Annex II, which the United Kingdom requested in 2007, and the decision from the EU Commission came only in 2011 (23).

Briefly, IVD devices placed in the EU market require a CE-mark to indicate conformity with the IVD Directive. For the high risk products listed in Annex II, the involvement of a Notified Body (NB) is required to assess conformity to the IVD Directive before placing the device in the European market. An NB is an organization accredited by a member state to assess the manufacturer’s conformity to the essential requirements of the directive.

Currently, any CDx assay entering the EU market is classified as low risk device based on a conformity assessment and CE-marking by the manufacturer, the so-called self-certification procedure. This results in incomprehensible differences in the regulatory pathway to the market between the USA (PMA approval) and the EU (self-certification).

However, there are major changes under way in EU IVD medical device legislation, which will impact CDx assays entering the market. The IVD Directive will be replaced by a Regulation on IVD (9). A regulation is the most powerful, single regulatory framework, which is applicable in a uniform manner at the same time for all EU member states, which leaves no room for divergent transpositions.

A draft of the new IVD Regulation (IVDR) has already been proposed, and obviously, we will be facing a very different regulatory landscape in the EU in the years to come (9). In the classification system proposed in the IVDR, IVDs will be assigned to four classification groups A, B, C, and D, depending on device risk, with class A being the lowest risk class. The four-class system resembles what we already know from the Canadian and Australian regulations, and is similar, but not equal, to what has been proposed by a Global Harmonization Task Force (24). CDx assays will be Class C devices and will require a complex regulatory pathway including a requirement for a Design Examination Certification by an NB. The review by the NB may possibly also be linked to a consultation with the European Medicines Agency (EMA) or, alternatively, compliance to a Common Technical Specifications (CTS) will be required. The CTS for new devices will be drafted as part of the review process. No matter which of the proposed pathways (EMA consultation or CTS) becomes final, the time to the market for a CDx assay will be extended essentially. It is assumed that the proposed IVDR will pass through the Council and Parliament in 2014, and the Regulation will then enter into force in 2017, after a 3-year implementation period.

### Japan expected to mirror FDA review and approval process

In Japan CDx assays are classified as high risk devices (class III), however, the regulatory approval process has until now been disconnected from the approval of the related therapeutic products. In October 2011, the Japan Association of Clinical Reagent Industries (JACRI) addressed the Ministry of Health, Labour and Welfare (MHLW) and the Pharmaceuticals and Medical Devices Agency (PMDA) with a proposal for a regulatory pathway for companion diagnostics (25). At the end of December 2013, the final guidance for CDx and related drugs was announced in the PMDA Notification. The guidance includes a CDx device definition, guidance for application for CDx and therapeutic products, clinical studies of therapeutic products as well as a review system by PMDA. In the original proposal presented to the MHLW/PMDA, JACRI has taken into consideration the FDA draft guidance on *In vitro* Companion Diagnostic Devices that was issued in July 2011 (8). Thus, the final PMDA guidance stresses that application for both a CDx and its corresponding therapeutic product should be submitted and reviewed at the same time under PMDA. Furthermore, it is recommended that drug and diagnostic sponsors seek early consultation with the authorities on the regulatory pathway for CDx, similar to the FDA Pre-Sub program. It is expected that publication of the guidance will improve the review process for companion diagnostics IVDs in Japan and make it more transparent. There is no English version of the guidance available at the moment on the PMDA website (26).

### China regulatory pathway is a challenge

Requirements for registration of IVD devices in China exceed requirements in any of the other countries and regions described above. Here, CDx assays are as in the US and Japan, classified as high risk devices (class III products). So far, there have been no guidance documents issued for CDx assays, but the registration process follows the requirements for class III products and requires extensive documentation and supporting testing data to be submitted to the China Food and Drug Administration. Specific for a class III IVD device in China, there is a requirement for local testing of at least 1000 patient specimens divided among three geographically distinct hospitals. This testing must be performed using three consecutive lots of the device and further detailed lot records, including specific requirements for stability testing and analytical performance testing must be provided. In addition, a number of legal documents are required to be submitted such as legal qualification of the manufacturer and authorization letters for authorized representatives.

## Conclusion and Future Considerations

Companion diagnostics holds the promise of improving the predictability of the oncology drug development process and become an important tool for the oncologist in relation to the choice of treatment for the individual patient. A number of drug–diagnostic co-development projects have already been completed successfully, and in the clinic, the use of several targeted cancer drugs are now guided by a CDx. For these drugs the management of the patient partly depends on the result generated by the CDx assay, and consequently this type of assay has become critical for the patient care. In order to avoid “false positive” and “false negative” test results, it must be documented for any CDx assay that it has a high degree of analytical and clinical validity (7). To some extent this is comparable to the safety and efficacy documentation that needs to be generated in order to achieve a marketing authorization for a new drug (27).

The central role of the CDx assays in relation to the current and future pharmacotherapy has attracted the attention of the regulators, especially the US FDA. In the US, CDx assays are in most cases classified as class III, high risk devices, for which the most stringent requirement for safety and effectiveness documentation apply, including submission of a PMA. Knowing the critical role of a CDx assay in relation to patient management this seems only reasonable that a number of other countries including Australia, Canada, China, and Japan have followed suit with regards to stringent requirements. However, for the EU, it has taken some time to realize the critical importance of CDx assays in relation to patient care and safety, and only recently the discussions about a more up to date regulation for IVD medical devices including CDx assay has started. Despite the coming new legislation in the EU not seeming to have the same formalized co-development and co-approval process as in the US, it will most likely increase the patient safety.

Many of the biological characteristics important for a specific drug to be effective, such as mutations, gene rearrangements, gene amplifications, and protein overexpression are typically not present in one cancer type alone, but are often found across several cancer diseases. *HER2* amplification and protein overexpression are such examples, where these characteristics are found in breast and gastric cancer as well as others cancers. Further, it has also been shown that an HER2 targeted drug like trastuzumab is effective in both breast and gastric cancer (3, 28). This and other examples have shown what matters most in relation to determining the response to a specific drug is the molecular pathways driving the growth of the cancer and not from where in the body the tumor originates. Based on this knowledge, we will probably see drug–diagnostic combination being developed for several cancer diseases simultaneously in the future, which will be both scientifically and medically challenging. How the drug regulatory system, such as the FDA, will handle this challenge will also be interesting to see, as both drugs and CDxs have been approved for one cancer disease at a time up to now.

Most of the CDx guided targeted cancer drugs that have been introduced within the last few years have shown significantly high response rates and prolonged progression free survival in specific selected groups of patients. Previously, for many of the treated patients no treatment has been available for their specific disease, and CDx guided drugs definitively represent a real progress within oncology. However, for all these drugs, resistance will develop at some point in time resulting in disease progression. For this reason it is unlikely that “monotherapy” with a targeted cancer drug based on identification of a single biomarker will achieve long-lasting remission, and we will probably need to move away from the “one biomarker one drug” model toward a more multimodal approach (29). This new model will need to integrate multiple biomarkers and multiple targeted cancer drugs and should be based on a simultaneous use of several drugs in order to block more signal pathways, thus to prevent resistance to develop. When it comes to CDx assays, this will make a call on specifically designed multiplex assays most likely based on technologies such as gene expression arrays or next generation sequencing (NGS) (30). Despite the very recent decision by the FDA to grant marketing authorization for the Illumina instrument platform for screening and diagnosis of cystic fibrosis, there still seems to be a number of challenges that must be overcome before we see NGS as CDx for targeted cancer drugs (31, 32). However, the advantages of this type of technology are that they will enable researchers and healthcare professionals to get a broader look at the cancer patients’ genetic makeup and probably help them designing more effective treatment modalities. Several CDx possibilities seem to be available to improve the treatment of the cancer patients, however, the development of assays will face a challenging time both with respect to medical/scientific as well as regulatory aspects.

## Conflict of Interest Statement

Jan Trøst Jørgensen is working as a consultant for Dako and has given lectures at meetings sponsored by Roche and AstraZeneca. Dana Olsen is an employee of Dako.
